# Supplementary material for: Structural basis for sequence-independent substrate selection by eukaryotic wobble base tRNA deaminase ADAT2/3
Source: Nat Commun. 2022 Nov 8;13:6737. doi: 10.1038/s41467-022-34441-z (PMC9643335; doi:10.1038/s41467-022-34441-z)
Supplement: Supplementary file 1 — Supplementary Information [file 41467_2022_34441_MOESM1_ESM.pdf]

Supplementary Information

Structural basis for sequence-independent substrate selection by eukaryotic wobble base tRNA deaminase ADAT2/3

Luciano G. Dolce[1], Aubree A. Zimmer[2], Laura Tengo[1], Félix Weis[3], Mary Anne T. Rubio[2], Juan D. Alfonzo[2] and Eva Kowalinski[1,*]

[1] EMBL Grenoble, 71 Avenue des Martyrs, 38042 Grenoble, France

[2]Department of Microbiology and The Center for RNA Biology, The Ohio State University, Columbus, Ohio, USA

[3] EMBL Heidelberg, Structural and Computational Biology Unit, Meyerhofstraße 1, 69117 Heidelberg, Germany

[*]Corresponding author email: kowalinski@embl.fr

Key words: ADAT; inosine; tRNA modification; deaminase; cryo-EM structure; *Trypanosoma brucei*

Supplementary Fig. 1: Biochemical characterization of *Tb*ADAT2/3.

(a) SDS-PAGE of fractions of the purified ADAT2 (24 kDa) and ADAT3 (43 kDa) complex. Experiment was repeated independently more than 10 times with similar results. **(b)** Thermostability assay of the ADAT2/3 heterodimer, with a melting temperature of 53.5 ˚C. **(c)** Different RNAs identified to co-purify with ADAT2/3 from overexpression in *E.coli*. The first graph represents the high abundant tRNA in comparison with all other RNA hits. The second represents the tRNA abundances compared to all tRNA identified, with $tRNA^{Thr}$ and $tRNA^{Pro}$ as the most abundant. **(d)** SEC-MALLS experiment with different molecular ratios of added tRNA, indicating a complex stoichiometry of 1:1:1 ADAT2:ADAT3:tRNA. **(e)** Model of ADAT2/3 as predicted by AlphaFold in cartoon representation. ADAT2 in salmon, $ADAT3^{CDA}$ in teal and $ADAT3^{N}$ in blue. Regions with IDDT lower than 50 were hidden from the representation. **(f)** Statistics from the AlphaFold model presented in E. **(g)** Superposition of the $ADAT2^{C}$ from the AlphaFold model (beige) and the final model (salmon). **(h)** RMSD between different regions of the AlphaFold model and the final model, as calculated by ChimeraX matchmaker tool.

Supplementary Fig. 2: Structure determination of *Tb*ADAT2/3 bound to tRNA by CryoEM.

(a) Example of a representative micrograph (resulting from the averaging of the motion corrected frames of a movie). **(b)** Patch CTF estimation from warp, where we see the relative tilt of the stage generating a defocus gradient in the micrograph. A more precise initial CTF estimation is of particularly importance in the case of tilted data collection. **(c)** First round of 2D classification used to separate the tRNA bound ADAT2/3 particles from the free tRNA or free ADAT2/3 particles. **(d)** First round of 3D classification to further remove free tRNA or free ADAT2/3 particles from our tRNA bound ADAT2/3 dataset. **(e)** Second round of 2D classification, to further remove low resolution particles. **(f)** Second round of 3D classification, to increase particle homogeneity. **(g)** Final refinement step, combining local CTF refinement with non-uniform refinement. **(h)** Post processing of the final refinement, performed by DeepEMhancer, that increased the connectivity of the map and allowed for the interpretation of the $ADAT3^{N}$ (colored as in figure 1, with ADAT2 in salmon, $ADAT3^{N}$ in blue, $ADAT3^{CDA}$ in teal and the tRNA in black). **(i)** Local resolution map calculated by CryoSPARC, displayed in Chimera, with level 0.1. **(j)** Final map GSFSC (Gold Standard Fourier Shell Correlation) as calculated by cryoSPARC indicates a resolution of 3.62A. **(k)** Angular distribution map of particle orientation as calculated by cryoSPARC.

Supplementary Fig. 3: Anticodon loop, active site and molecular RY gate.

(a) Anticodon loop of the tRNA, with EM density as a blue mesh. Hydrogen bond of the non-canonical base pair between C_{32} and A_{38} in cyan. **(b)** Active site with EM density as blue mesh. **(c)** Comparison with the catalytic site of the yeast crystal structure (PDB: 7BV5). Water molecule in red, zinc atom in yellow, dotted lines representing the zinc coordination, side chains colored by heteroatom. **(d)** Gate residues R159 and Y205 of ADAT2. Absence of density for the side chain of R159 suggests its flexibility. **(e)** Base U_{36} is sandwiched through π-π stacking with base C_{32} and gate residue Y205. **(f)** Detail of the closed conformation of the molecular RY gate as in the structure of *Mm*ADAT2/3 (PDB: 7NZ7). Electron density map contoured at 1 σ as blue mesh. **(g & h & i)** Comparison of an ADAT3 conserved loop between eukaryotic bound and unbound structures: **(g)** *Tb*ADAT2/3; **(h)** *Mm*ADAT2/3 (PDB: 7NZ7), with overlay of the tRNA from the *Tb*ADAT2/3 structure for visualization purposes; **(i)** bacterial RNA-bound (*Sa*TadA, PDB: 2B3J). All RNA in black, and side chain and nucleosides colored by heteroatom. **(j & k)** Single turnover assays of wild-type *Tb*ADAT2/3 and selected *Tb*ADAT2 gate mutants co-expressed with wild-type *Tb*ADAT3. A_{34} to I_{34} conversion was measured over 1 h in the presence of excess enzyme; except for $R159Y^{TbADAT2}$, which was measured over 4 h. The dashed line in K represents the maximum A-to-I fraction in the wild-type assay. The fraction of inosine formed was plotted against time and fit to a single exponential curve [$f = a(1 - e^{-kt})$], where f represents product

formed, a denotes product formed at the end point of the reaction, k signifies $k_{obs,}$ and t is time. Data are presented as mean values and error bar as standard deviation. n = 3 independent experiments for wt and R159Y constructs, n = 5 for Y205F, and n = 7 for R159K. Source data are provided as a Source Data file. **(l)** Circular dichroism spectra of wt and different ADAT2/3 gate mutants. EM densities in this figure derived from the final non-b-factor-sharpened cryoSPARC map.

Supplementary Fig. 4: The N-terminal domain of ADAT3 is involved in tRNA recognition and binding.

(a) Alignment of ADAT3 N-terminal domains, with the sequences for *trypanosoma brucei*, human, mouse and yeast. Structural elements predicted by AlphaFold for *Tb*ADAT3^N are annotated with alpha-helices as cylinders and beta-sheets as arrows. Residues identified as important for tRNA binding or deamination in the corresponding species are annotated as olive triangles above for *Mm*ADAT3^N and yellow triangles below for *Sc*ADAT3^N.The annotated blue box indicates a kinetoplast specific region, which is predicted to be disordered. Other colors and boxes in the alignment represent relative conservation above 70%. **(b)** Comparison between the CryoSPARC non-uniform refinement map and the DeepEMhancer post-processed map of ADAT2/3, focused on the ADAT3^N region, with the model cartoon, and the residues identified as important for tRNA binding indicated. Maps and models colored as in figure 1. **(c)** Example gels of EMSA of different ADAT2/3 constructs. **(d)** Quantification of the unbound fraction of tRNA in the EMSA. Data are presented as mean values and error bar as standard deviation. n = 3 independent experiments. Source data are provided as a Source Data file . **(e)** SEC chromatogram of different ADAT2/3 constructs used in the tRNA binding assay. **(f)** SDS-PAGE showing different ADAT2/3 constructs sample after SEC. Experiment was repeated independently two times with similar results.

Supplementary Fig. 5: Comparison between *Sa*TadA and *Tb*ADAT2/3 interaction with tRNA.

(a & b) Schematic overview of the anticodon stem loop region of the tRNA and its interactions with **(a)** *Sa*TadA and **(b)** *Tb*ADAT2/3, respectively. Zinc coordinating sites are highlighted in yellow. ADAT2 residues are represented in salmon, ADAT3 residues in teal. Residues of the TadA α-protomer of the homodimer (corresponding to ADAT2 relative to the ligand) in grey and residues of the β-protomer (corresponding to the ADAT3 relative to the ligand) in pink. **(c & d)** Schematic of the whole tRNA with interaction contacts for *Sa*TadA and *Tb*ADAT2/3. Anti-codon stem loop contacts are represented as a summary of the ones in A and B**.** Since no structure of TadA bound to full length tRNA is available, the same schematic tRNA is used for both. Colors as in A and B.

Supplementary Fig. 6: ADAT3 pseudo-active site plug and comparison of ADAT3^N position.

(a, b, c & d) Comparison of the pseudo active sites plug in **(a)** *Tb*ADAT3 (with coulomb density as blue mesh), **(b)** *Mm*ADAT3 (PDB: 7NZ7), and **(c)** *Sc*ADAT3 (PDB:7BV5). **(d)** *Tb*ADAT2 active site as a comparison. Zinc atom in yellow, dotted lines representing the zinc coordination, side chains colored by heteroatom. All ADAT3 sites are obstructed by ADAT3 protein loops ("plugs"). **(e & f)** Sequence alignment of kinetoplast, mammalian and yeast ADAT3s regions representing the "plug" protein loops. The residues in yellow boxes correspond to the plug in each eukaryotic clade. A gray star represents the reaction inert residue that substitutes the glutamic acid in the active site. The plug is present in clade specific loops or non-conserved regions, indicating that this feature has divergently evolved. Colors and boxes in the alignment represent relative conservation above 70%. **(g)** Expression and purification test of wild-type *Tb*ADAT2 / wild-type *Tb*ADAT3 in comparison with wild-type *Tb*ADAT2 / *Tb*ADAT3 Y236 mutants. All tested point mutations lead to insoluble protein reaffirming the importance of Y236 as acquired additional Zn^{++} coordinating residue. Total fraction after lysis of the insect cells; soluble fraction after the lysate

clarification; elution from the Ni-NTA resin capture. Experiment was repeated independently two times with similar results. **(h)** Comparison of ADAT3^{N} position in our model and the apo crystal structures *Mm*ADAT3 (in olive, PDB: 7NZ7), and *Sc*ADAT3 (in yellow, PDB:7BV5), represented as surface and aligned in ChimeraX using the CDA domain as reference. In magenta we compare the position of ADAT3^{N} with the D1 domain of *Mj*Trm5 (PDB: 2ZZM), which binds to the tRNA in a similar way, but in a slightly different position and was previously used as a reference to study the ADAT^{N} interaction. tRNA in black.

Supplementary Fig. 1

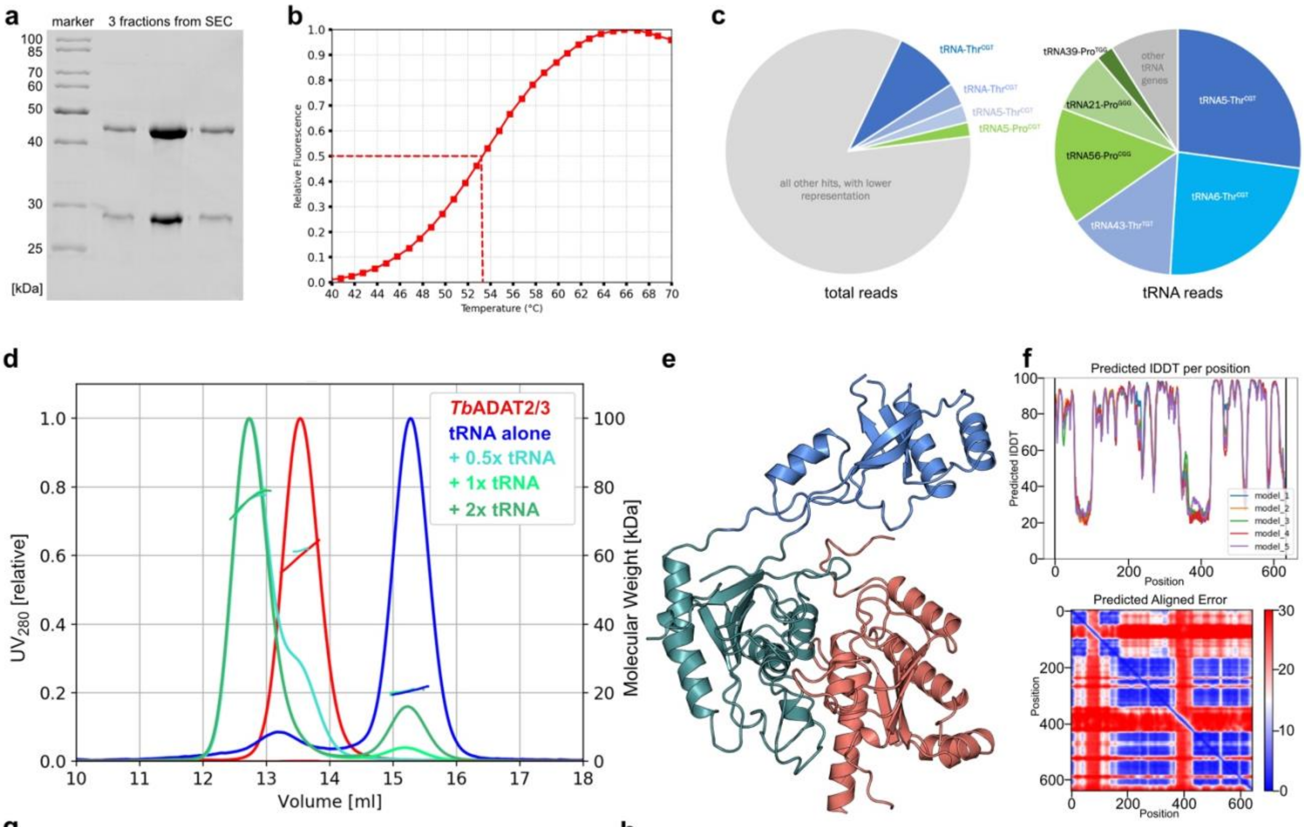

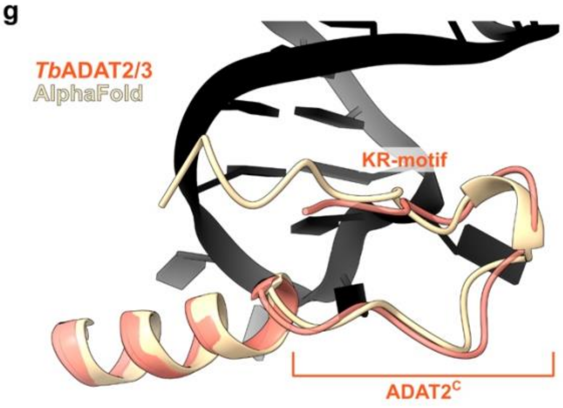

h

Comparison between the AlphaFold model and the final model

Region	RMSD (A)
ADAT2^{CDA}	0.907
ADAT2^{C}	1.296
ADAT3^{CDA}	0.766
ADAT3^{N}	1.078

Supplementary Fig. 2

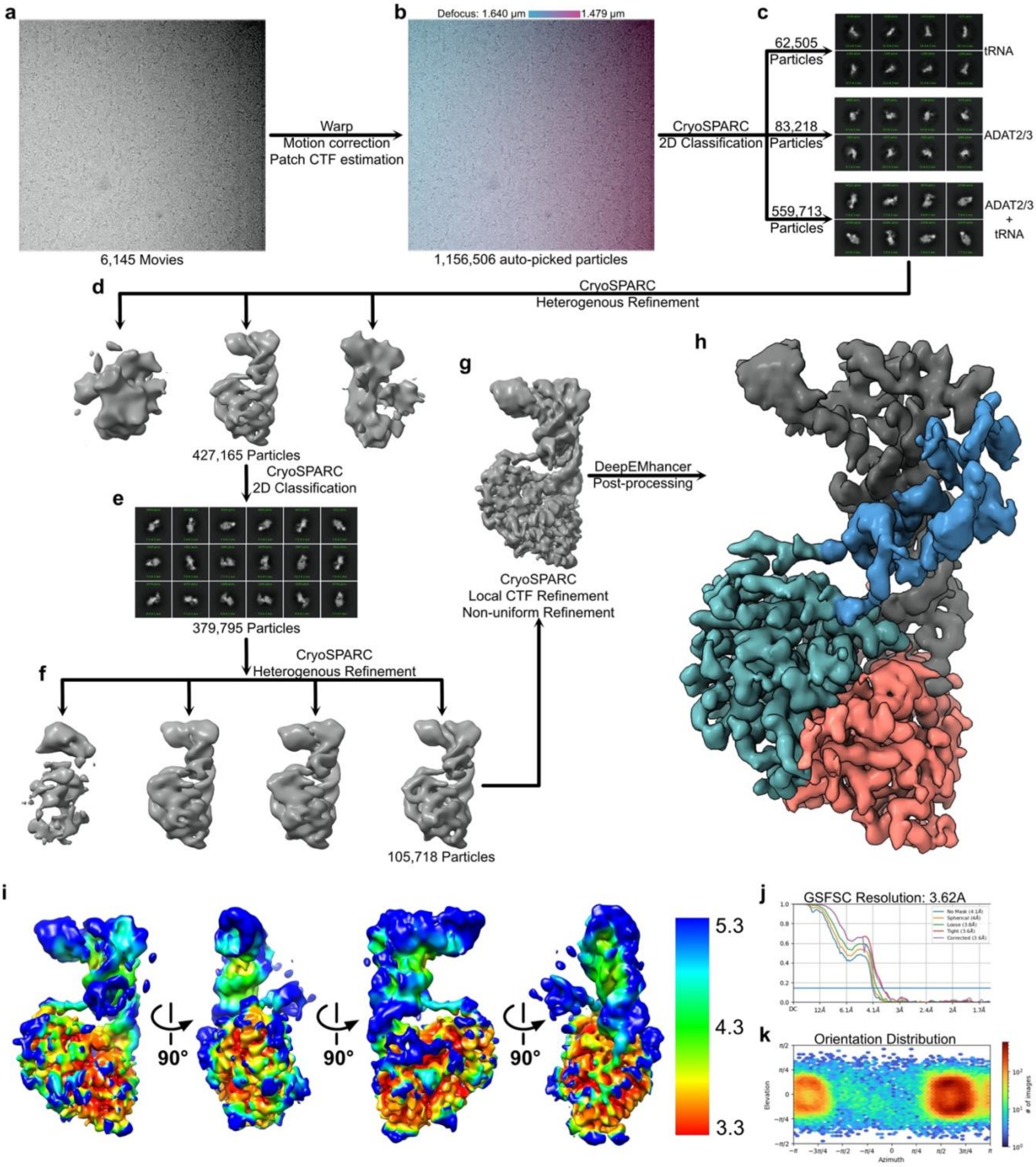

Supplementary Fig. 3

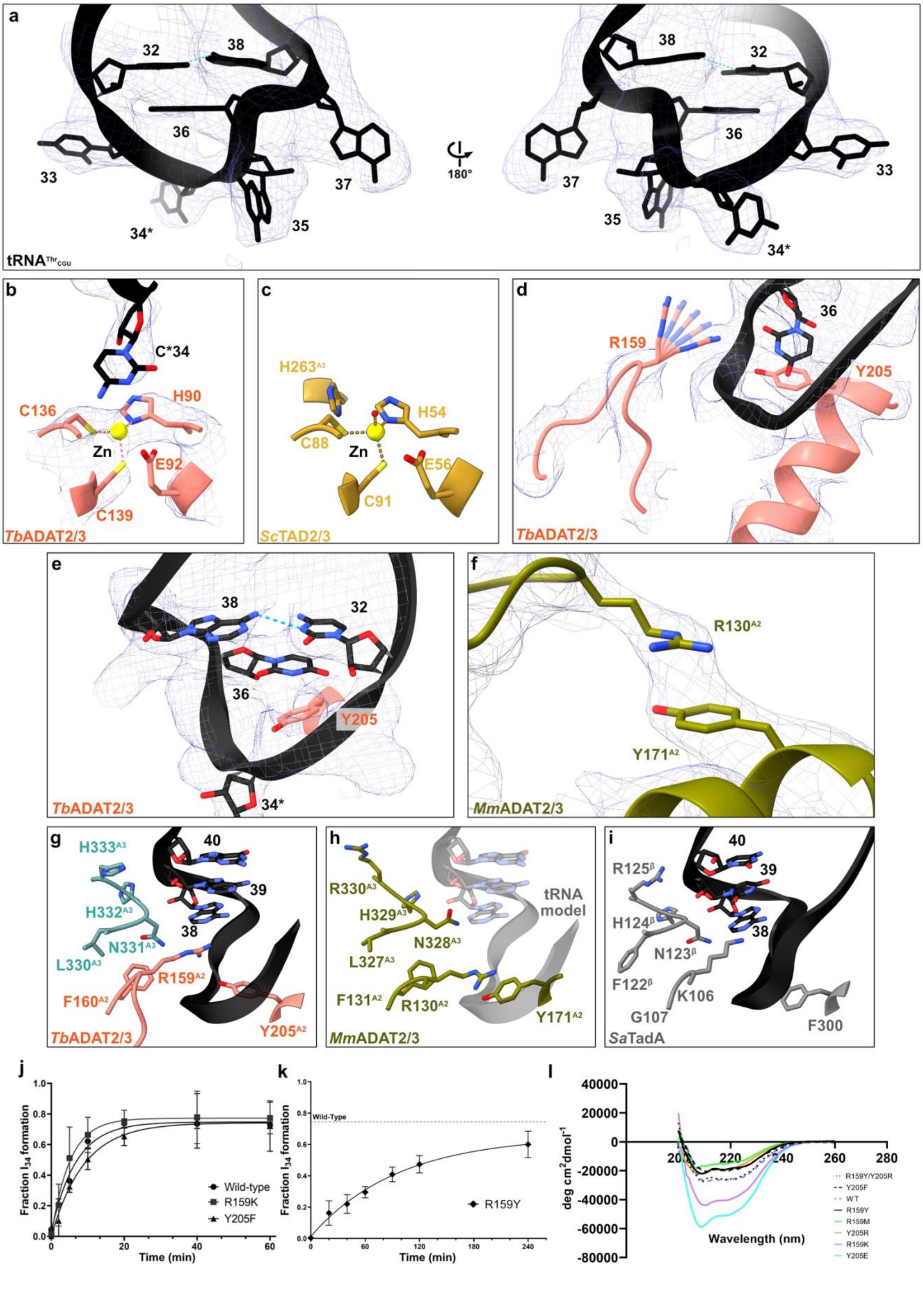

Supplementary Fig. 4

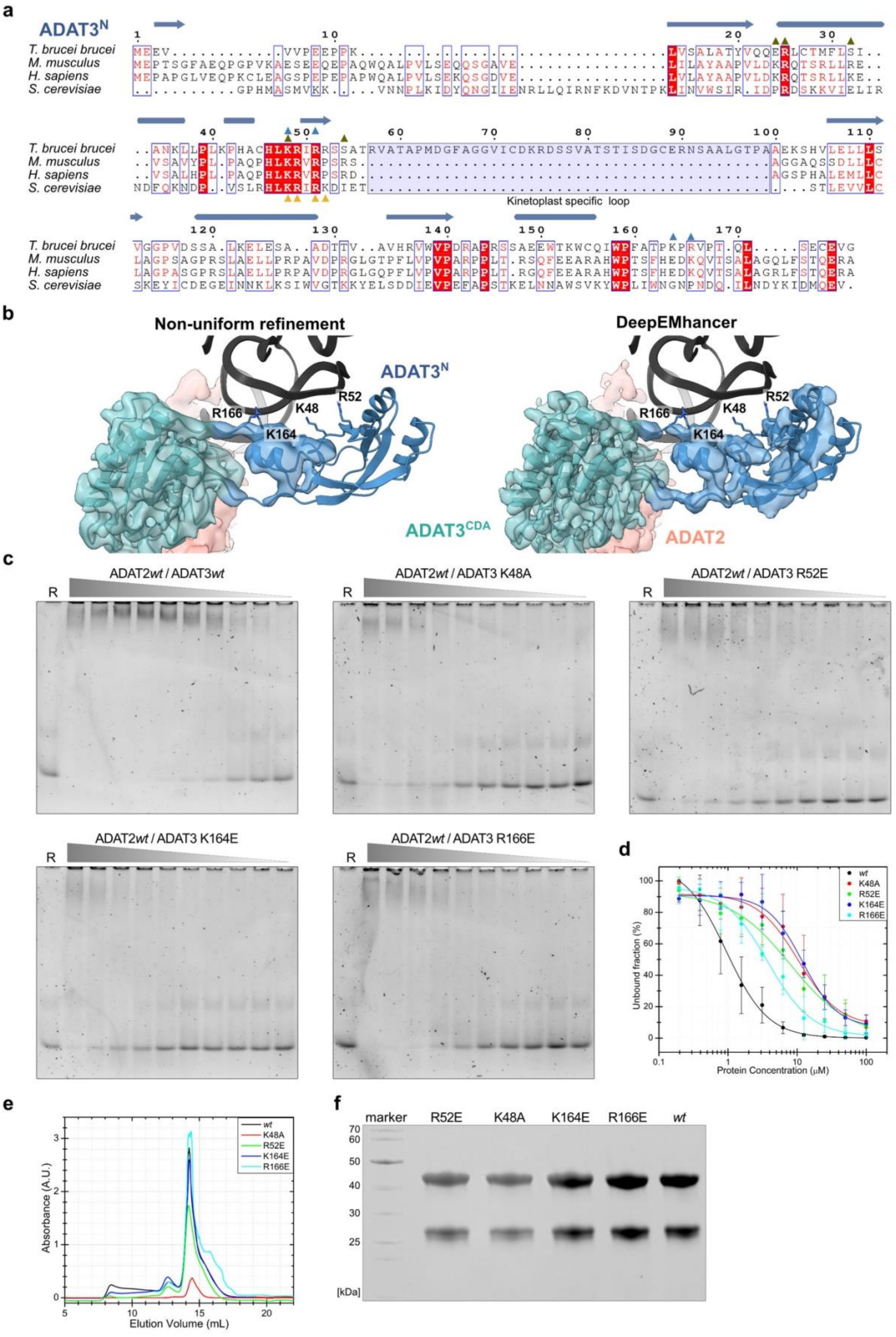

Supplementary Fig. 5

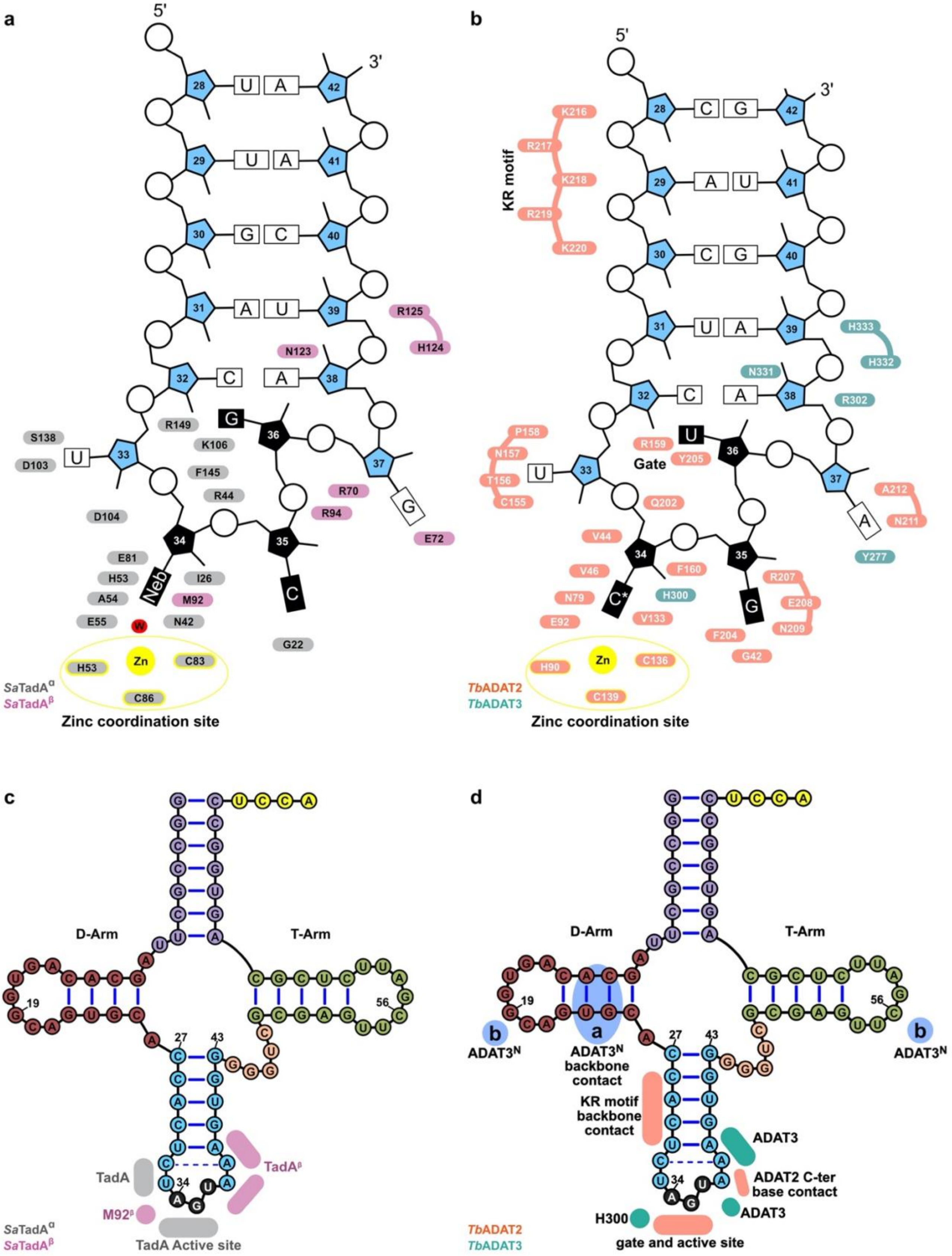

Supplementary Fig. 6

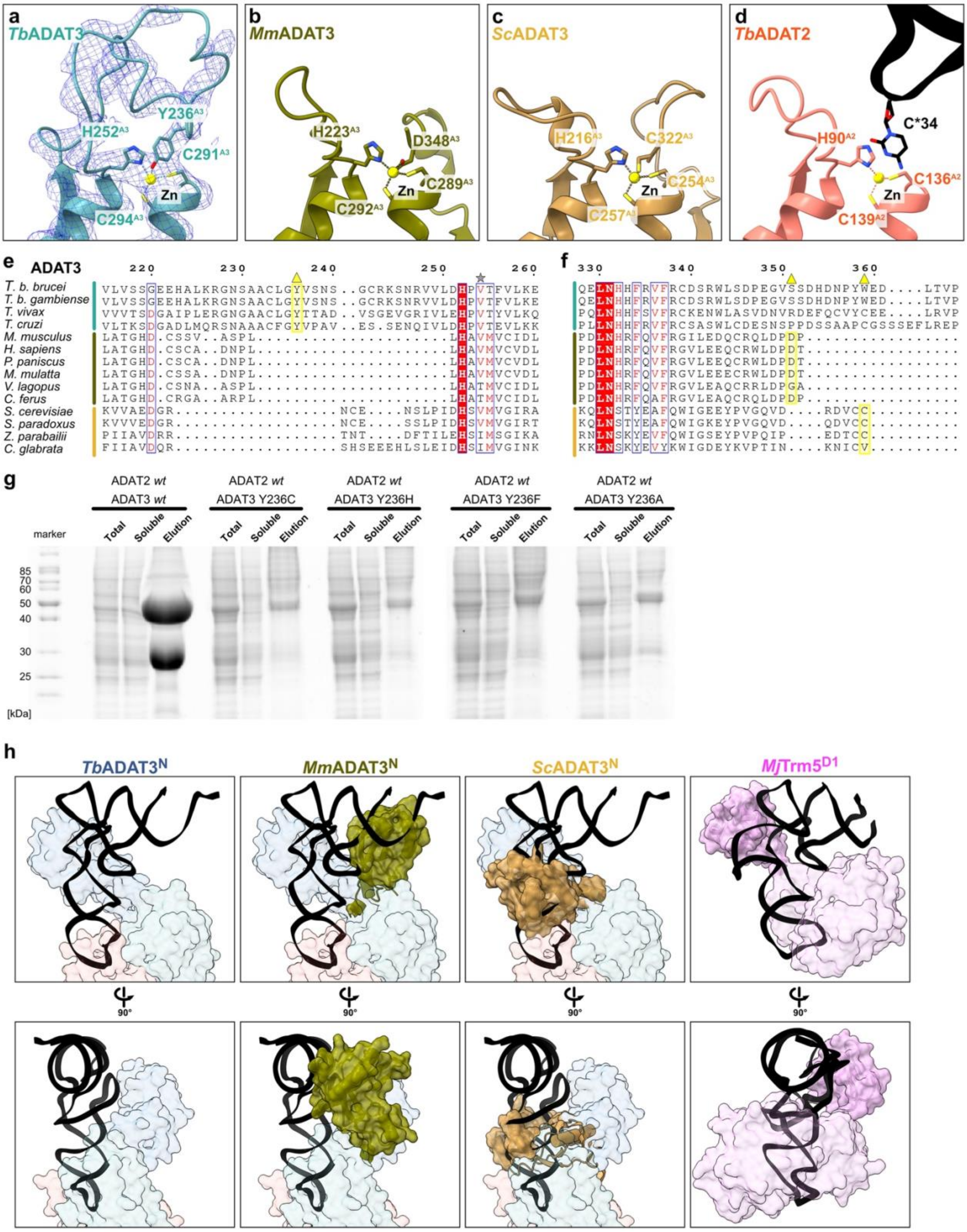

Supplementary Table 1. Summary of Cryo electron microscopy data collection and refinement statistics	
Data Collection	tRNA bound *Tb*ADAT2/3
Microscope	Titan Krios
Voltage	300 kV
Camera	Quantum-K2
Energy filter	GIF
Spot size	8
Beam size	650 nm
Pixel size (A/pix)	0.81
Preset defocus range	-1.0 to -1.8
Stage tilt	30 deg
Dose rate (e^-/pixel.s) / total exposure (e^-/A^2)	3 / 55
Number of frames per movie	80
Number of movies	6,145
Data Processing	
Initially warp autopick particles	1,156,506
Final number of particles	105,718
Resolution FSC	3.62
Sharpening method	DeepEMhancer
EMDB accession number	EMD-15690
Refinement	
PDB accession number	8AW3
No atoms	5155
Residues (protein)	459
Residues (RNA)	76
CC_{box}, CC_{mask}, CC_{volume}	0.69, 0.65, 0.67
R.M.S.D.	
Bond lengths	0.003
Bond angles	0.651
Ramachandran favored (%)	94.85
Ramachandran allowed (%)	5.15
Ramachandran outlier (%)	0
MolProbity score	2
Clash score	13.77

Supplementary Table 2. Observed reaction rate for wild type and RY molecular gate mutants	
Enzyme	K_{obs} (min^{-1})
WT	0.148 ± 0.002
ADAT2 Y205E	n.d.
ADAT2 Y205R	n.d.
ADAT2 Y205F	0.15 ± 0.04
ADAT2 R159M	n.d.
ADAT2 R159Y	0.010 ± 0.001
ADAT2 R159Y/Y205R	n.d.
ADAT2 R159K	0.3 ± 0.1

All mutants are of T. brucei ADAT2 co-expressed with TbADAT3.
n.d. denotes no detectable activity after 24-hour assay incubation.
K_{obs} values obtained from single turnover kinetic assays (n=3).

Supplementary Table 3. Summary of published tRNA binding mutants in the ADAT3 N-terminal domain and their corresponding residues in *T.brucei*		
$ADAT3^N$ mutant	Effect	Corresponding *Tb* residues
*Sc*K72A/R73A	tRNA interaction defect (Liu et al., 2020)	K48/R49
*Sc*R75A/K76A	tRNA interaction defect (Liu et al., 2020)	R51/R52
*Mm*K53E/R54E/R61E	Deamination defect (Ramos-Morales et al., 2021).	E24/R25/S32
*Mm*K53E/R54E/R61E/K76E/R82E	Deamination defect (Ramos-Morales et al., 2021).	E24/R25/S32/K48/S54
